# Aflatoxins in Mexican Maize Systems: From Genetic Resources to Agroecological Resilience and Co-Occurrence with Fumonisins

**DOI:** 10.3390/toxins17110531

**Published:** 2025-10-29

**Authors:** Carlos Muñoz-Zavala, Obed Solís-Martínez, Jessica Berenice Valencia-Luna, Kai Sonder, Ana María Hernández-Anguiano, Natalia Palacios-Rojas

**Affiliations:** 1 Colegio de Postgraduados (COLPOS), Campus Montecillo, Texcoco 56264, Mexico; 2 International Maize and Wheat Improvement Center (CIMMYT), Texcoco 56237, Mexico; k.sonder@cgiar.org; 3 National Institute of Public Health (INSP), Cuernavaca 62100, Morelos, Mexico; obed.solis@insp.edu.mx

**Keywords:** *Aspergillus flavus*, *Fusarium verticillioides*, mycotoxin, atoxigenic strains, landrace, agroecological practices

## Abstract

Aflatoxins (AFs) and fumonisins (FUMs) are among the most prevalent and toxic mycotoxins affecting maize production globally. In Mexico, their co-occurrence poses a significant public health concern, as maize is not only a dietary staple but also predominantly grown and consumed at the household level. This review examines the multifactorial nature of AFs and FUMs contamination in Mexican maize systems, considering the roles of maize germplasm, agricultural practices, environmental conditions, and soil microbiota. Maize landraces, well-adapted to diverse agroecological zones, exhibit potential resistance to AFs contamination and should be prioritized in breeding programs. Sustainable agricultural practices and biocontrol strategies, including the use of atoxigenic *Aspergillus flavus* strains, are presented as promising interventions. Environmental factors and soil characteristics further influence fungal proliferation and mycotoxin biosynthesis. Advances in microbiome engineering, biological breeding approaches, and predictive modeling offer novel opportunities for prevention and control. The synergistic toxicity of AFs and FUMs significantly increases health risks, particularly for liver cancer, highlighting the urgency of integrated mitigation strategies. While Mexico has regulatory limits for AFs, the lack of legal thresholds for FUMs remains a critical gap in food safety legislation. This comprehensive review underscores the need for biomarker-based exposure assessments and coordinated national policies, alongside multidisciplinary strategies to reduce mycotoxin exposure and enhance food safety in maize systems.

## 1. Introduction

### 1.1. Maize in Mexico and the World

Maize (*Zea mays* L.) is of worldwide importance as food, feed, and a source of diverse industrially important products. It is also of cultural relevance, especially in Latin America and Africa, and is a model genetic plant with a vast genetic diversity. Although maize was first domesticated in Mexico, in the mid-elevations of South-Central Mexico, and occurred with the teosinte (*Zea mays* ssp. Mexicana (Schrader) Iltis) race Balsas [1], maize was then introduced to different continents, including North and South America, Europe, Africa, and Asia. The Food and Agriculture Organization of the United Nations (FAO) estimates a production of 1220 million metric tons (Mt) of maize grain by 2025. Top producing 10 countries are United States (31%), China (24%), Brazil (11%), European Union (5%), Argentina (4%), India (3%), Ukraine (2%), Mexico (2%), South Africa (1%) and Canada (1% ) [2].

Estimated maize production in Mexico for 2025 is expected to be approximately 24.4 Mt, while imports are projected at 21.6 Mt, which is provided from the United States, Argentina, Brazil, and Canada [3]. White maize accounts for 86.9% of the production, which is self-sufficient in domestic production in all 32 states of the country. The main producing states are Sinaloa, Jalisco, the State of Mexico, and Guanajuato. White maize is mainly used for human consumption, providing 30% of the protein and 40% of the energy in the diets of consumers [4]. Yellow maize accounts for 13.1% of production in the states of Chihuahua, Jalisco, and Tamaulipas, with a national deficit of 80% that is covered by imports and is mainly used in industry and for animal feed [3]. The total seed use in Mexico corresponds to 57.5% of maize landraces, and the rest corresponds to improved (hybrid maize) [5]. A total of 67% of maize landrace production is primarily for self-consumption, while the remainder is for local marketing, although the percentages vary depending on the region and local agricultural practices. Seven specific agroecosystems have been identified as priority areas for the in situ conservation of 68 local maize varieties, which have been cultivated and adapted for centuries by local farming communities in Mexico [6]. Although genetic diversity has been used to develop stress-resilient germplasm, studies to identify resistance to mycotoxin-producing fungi are still very limited [7].

### 1.2. Climate Change and Mycotoxins of Major Relevance in Maize

**Figure 1:**
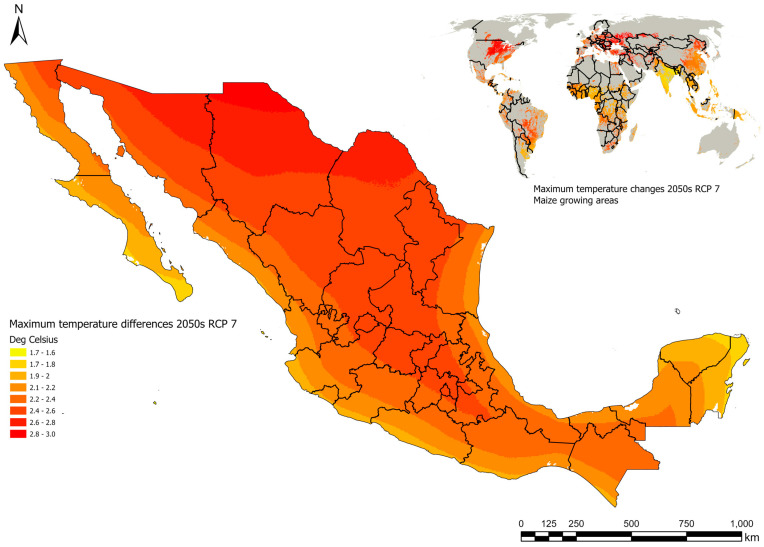
Map of the predicted maximum temperature changes for the 2050s under RCP 7 CMIP6 in Mexico and for maize growing areas globally. The most vulnerable maize-producing regions (red regions) are due to changes in temperature increases that will negatively impact susceptibility, production, and mycotoxin contamination.

The impact of climate change (CC) on agricultural production is greatest in the tropics and subtropics, where temperature and humidity favor the development of plant diseases, extreme droughts, and heat. Regions with temperate climates may become more vulnerable to production losses due to the risk of mycotoxin contamination [8]. Studies based on data from 186 countries worldwide between 1980 and 2020 have shown that Mexico is among the countries that will be most affected by CC [9]. Both changes in precipitation (https://ars.els-cdn.com/content/image/1-s2.0-S0168169925002467-gr7_lrg.jpg, accessed on 26 June 2025) and temperature increase will have a negative impact on yields in the country [10,11]. The most vulnerable maize-growing zones are the non-irrigated production zones (Jalisco, Guanajuato, Michoacán, Puebla, and Zacatecas) and the coastal areas of the Pacific Ocean (Chiapas, Guerrero, Oaxaca, Sinaloa, and Sonora), the Gulf of Mexico (Tamaulipas and Veracruz), and the Yucatán Peninsula (Campeche and Quintana Roo) (Figure 1) [12,13].

Although more than 60 years have passed since the isolation and characterization of the first mycotoxin, challenges are increasing due to CC, genetic changes in fungi, and unforeseen effects of crop improvement, including its genetic makeup, protein expression, and metabolite levels [14]. Mycotoxins are the cause of economic losses and have great implications for the health and nutrition of consumers. They can induce hepatotoxicity, immunotoxicity, neurotoxicity, and nephrotoxicity in humans and other animals [15]. Globally (including Mexico), two highly relevant mycotoxins are produced in maize by *Fusarium* and *Aspergillus* species, FUMs and AFs, respectively [16]. FUMs are the most common in maize, produced mainly by *Fusarium verticillioides*, followed by *F. proliferatum* or in combination. FUMs are split into four groups identified as A, B, C, and P; group B includes the four most active FUMs (B1, B2, B3, and B4). In particular, FUMB1 is of greatest concern because it has been linked to leukoencephalomalacia in horses, edema in pigs, and liver and esophageal cancer in humans [17]. It is classified by the International Agency for Research on Cancer (IARC) as a possible class 2B human carcinogen [18]. However, AFs are the most harmful and are produced by some specific strains of *Aspergillus flavus*, *A. parasiticus*, and *A. nomiae*, with *A. flavus* being the most common. Among the four major AFs (B1, B2, G1, and G2), AFB1 is the most prevalent and potent because it can bind to DNA and modify its structure, inducing genotoxicity and mutagenicity [19]. The IARC has classified AFB1 as a Group 1 carcinogen for humans [20]. AFB1 undergoes metabolic processes in the liver, facilitated by CYP1A2 and CYP3A4 enzymes, leading to the formation of various metabolites, including aflatoxin M1 (AFM1) in milk and urine [21].

Globally, mycotoxin contamination in raw and processed cereals (maize, soybeans, wheat, barley, and rice) has been monitored in 100 countries across 15 geographic regions (including Mexico) over a 10-year period (2008–2017). In the analysis of 74,821 samples, 88% were found to be contaminated with at least one mycotoxin, and of these, 64% were contaminated with ≥2 mycotoxins [21]. In the case of maize, the most frequent combination was AFs and FUMs, with varying levels of contamination in each region and year due to climatic conditions (precipitation and temperature) during the sensitive periods of flowering and grain development. In Latin America, during 2023, 17,565 maize samples were evaluated from Argentina (*n* = 654), Bolivia (*n* = 110), Brazil (*n* = 15,895), Colombia (*n* = 89), El Salvador (*n* = 25), Ecuador (*n* = 74), Mexico (*n* = 132), and Peru (*n* = 586) were evaluated. Overall, the results revealed a high prevalence of FUMs and a low to moderate prevalence of AFs [22]. Although FUMs and AFs can be degraded during the nixtamalization process and other food processes used in the region, the elimination of mycotoxins cannot be guaranteed if the grain is heavily contaminated, and also because in Mexico, there is a high consumption of tortillas and other products derived from nixtamalized maize [23].

### 1.3. Regulation of Aflatoxins and Fumonisins

The effects of mycotoxins on humans and animals have led several countries to establish maximum acceptable limits for AFs and FUMs through various international institutions, including the U.S. Food and Drug Administration (FDA) and the European Food Safety Authority (EFSA), to protect human health and the economic interests of producers and trade. In Mexico, the Official Mexican Standard NOM-188-SSA1-2002 on Products and Services established the maximum level of AFs allowed in cereals intended for human and animal consumption, as well as the sanitary requirements for the transport and storage of these products (Table 1) [22]. However, no specific regulations exist for FUMs in grains. This regulatory deficit may be due to the limited number of studies and the complexity of FUM contamination. In this case, the FDA maximum limits were used as a reference for grain commercialization in Mexico [23].

Given the importance of maize production and consumption and the impact of CC in Mexico, this review explores the potential of germplasm type (landrace and hybrid maize), agricultural practices, and environmental conditions in the mitigation of AFs in maize, suggesting sustainable strategies adapted to the tropical and subtropical agroecological zones of Mexico. This is considering the co-occurrence of FUMs.

## 2. Types of Germplasm and the Aflatoxin Production

### 2.1. Advances in Resistance Development

In maize, good ear coverage, followed by cutin and waxes present in the pericarp and seed coat, can serve as a physical barrier to prevent fungal entry and avoid AF biosynthesis after infection [24]. At the cellular level, the AFs’ resistance mechanism is a complex quantitative trait that may be governed by multiple genes in an interaction of numerous anti-oxidant compounds by additive gene effects [25]. Most breeding programs aiming to develop disease resistance are led by the private sector; however, to our knowledge, they have made limited efforts to develop AF-resistant maize varieties. In contrast, public sector breeding programs have achieved significant progress in improving resistance to *Aspergillus* ear rot [26]. A direct correlation between fungal colonization and AF accumulation is not always observed, suggesting the potential involvement of host genes that disrupt or suppress the AFs’ biosynthetic pathway [27]. Table 2 lists tropical maize inbred lines reported to be resistant to *A. flavus* and associated with reduced AF accumulation that have been evaluated in other countries and could be tested in Mexico. Major quantitative trait loci (QTLs) for resistance are frequently identified in lines derived from these genetic backgrounds. To date, over 15 QTLs have been reported, with the region at bin 4.08 consistently showing a moderate but stable phenotypic effect. [28]. In other studies, bins 2.04, 4.05, and 8.03 were identified through a comprehensive meta-analysis of QTL and transcriptome data [29]. Recently, a specific gene, *Zm00001d021197*, selected during the domestication of teosinte to modern maize, was found to play a role in cell membrane formation and possess alpha-L-fucosidase activity, promoting glycoside metabolism and contributing to polysaccharide degradation, releasing sugars for microbial utilization, potentially alleviating stress caused by *A. flavus* [30].

Studies on maize hybrids with higher concentrations of beta-carotene (BC), beta-cryptoxanthin (BCX), and provitamin A (total proVA) have demonstrated significantly lower AF contamination than that in grains from hybrids with lower carotenoid concentrations [46,47]. This suggests that some carotenoids can be used as a component of strategies to combat AF contamination problems in maize and vitamin A deficiency.

### 2.2. Resilience and Susceptibility to Aflatoxins in Maize Landrace

Maize landraces (MLR) in Mexico exhibit significant variation in their susceptibility to AF contamination. During 2006 and 2008, seventy-four MLR accessions were collected in the central-western and north-western regions of Mexico to evaluate both *A. flavus* reproduction and AF contamination [48,49,50]. The Tabloncillo (MLR 2006-23) and Vandeño (MLR 2007-06) races are the two potential sources of resistance for maize breeding (Table 3), as they allow less reproduction of the fungus during long storage periods, but their resistance mechanisms remain unknown [48,49,50]. The MLR Tuxpeño is the progenitor or a major contributor to most of the resistant lines and probably the source of resistance. However, other sources could allow the incorporation of new MLR resistance into future breeding programs. Genetic characterization of parentage, kinship, diversity, and population substructure will enable the use of this resource for mapping AFs’ resistance associations and identifying the underlying factors contributing to this complex and challenging quantitative trait [51].

### 2.3. Hybrid Maize and Its Relationship with the Presence of Aflatoxins

Research on hybrid maize and AF contamination has revealed complex relationships influenced by environmental factors, fungal presence, and genetic resistance. Hybrids that are neither adapted to the region of planting nor drought-tolerant tend to be more susceptible to AFs [52]. Research efforts have led to the identification of commercial hybrids with resistance to AFs and good grain yield potential, with some hybrids showing AF values below 20 µg/kg in northeastern and southeastern Mexico (Table 3) [53]. However, multiple mycotoxin contamination remains a concern, as studies have detected several mycotoxins in treated maize seeds and grains [54].

**Table 3:** Description of two maize landrace accessions and seven commercial hybrids resistant to *A. flavus* reproduction and aflatoxin contamination in Mexico.

Maize Code	Name	Characteristics	Current Distribution (State Level)	Reference
**Landrace maize**				
MLR 2006-23	Tabloncillo	Elongated cobs with jagged or semicrystalline grains varying from white to orange	Michoacán, Jalisco, Nayarit, Sinaloa, and Sonora	[48,49,50]
MLR 2007–06	Vandeño	Cylindrical cobs with a thick ear and white jagged grains	Chiapas, Oaxaca and Guerrero	[48,50]
**Tropical white maize hybrid**				
NB-722	Novasem	Excellent stability, adaptability, and *Fusarium* tolerance	Tamaulipas	[53]
AG-2525	Anzu	Cob health and high yields	Tamaulipas	[53]
P-3057	Pioneer	Early maturity, strong stalks, and high yields	Tamaulipas	[53]
CORONEL	Iyadilpro	Excellent plant health, good ear coverage, and tolerance to stalk lodging	Campeche	[53]
P-4028	Pioneer	Good foliar and grain health	Campeche	[53]
P-4279	Pioneer	Good foliar and grain health	Campeche	[53]
TORNADO	Ceres	Excellent plant health and tolerance to stalk lodging	Campeche	[53]

## 3. Agricultural Production Systems and Aflatoxin Incidences

### 3.1. Impact of Agronomic Practices on Aflatoxin Contamination in Grain

Aflatoxin (AF) contamination in maize typically originates in the field, when *Aspergillus flavus* infects developing ears under warm conditions, particularly in the presence of drought stress and insect damage. In subtropical and tropical regions of Mexico, maize is usually grown in two annual cropping cycles: spring–summer (SS), predominantly rainfed (approximately 70%), and autumn–winter (AW), largely irrigated. However, farmers commonly face challenges such as limited rainfall and irrigation water, rising costs of inputs (e.g., seeds, electricity, fuel), and constrained access to credit. Although weather conditions in SS2024 improved from the previous year, prolonged drought and macroeconomic conditions remain challenging for Mexican farmers [55]. Intensive maize cultivation practices can significantly affect AF contamination levels in small-scale agricultural areas. Studies in different regions have revealed variable contamination rates influenced by farming practices and environmental conditions.

Agroecological practices in Mexican maize production—such as conservation agriculture [56], diversified crop rotations with cover crops [57,58], and traditional *milpa* systems involving crop associations—may offer viable alternatives to mitigate risks associated with climate change (CC), including the increased incidence of mycotoxin-producing fungi. Table 4 compares the conventional (intensive) practices with integrated crop management (agroecological) practices, highlighting their challenges and presenting that agroecological practices are the best practices for reducing mycotoxin contamination for achieving sustainable maize production while maintaining a cleaner and safer environment.

### 3.2. Environmental and Edaphic Factors Associated with Aflatoxin Incidence

**Figure 2:**
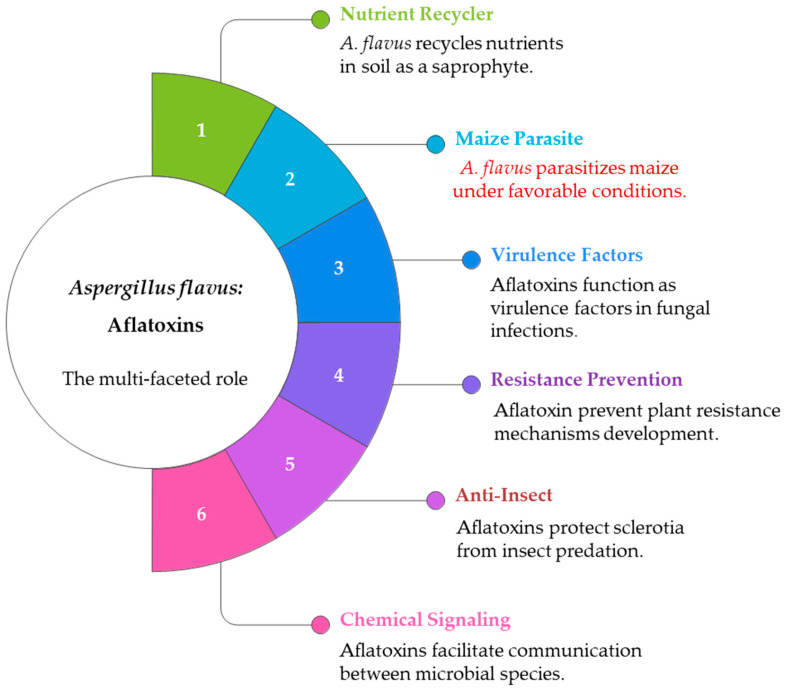
The multifaceted role of *A. flavus* and aflatoxin production in nature and agriculture.

*A. flavus* grows as a saprophyte in the soil, where it plays an important role as a nutrient recycler, supported by plant and animal residues [60]. Under favorable conditions (warm days and absence of rainfall), *A. flavus* can exist as sclerotia (resistant structures) or mycelia (fungal body) and parasitize susceptible maize [61]. During infection and colonization, the fungus forms conidiophores (ramification of the fungus), from which conidia (asexual spores) are released and transported by wind from the soil to plants. In nature, *A. flavus* produces AFs as a virulence factor to prevent the development of resistance mechanisms in plants, as an anti-insect agent to protect sclerotia from insect predation, and as a chemical signal between species (Figure 2) [17,60,61]. This process can be reinforced by damage caused by *Spodoptera frugiperda*, *Helicoverpa zea*, *Fusarium verticillioides*, and *Sitophilus zeamais*, as well as birds and rodents, which provide entry sites for *A. flavus* (Table 5).

**Figure 3:**
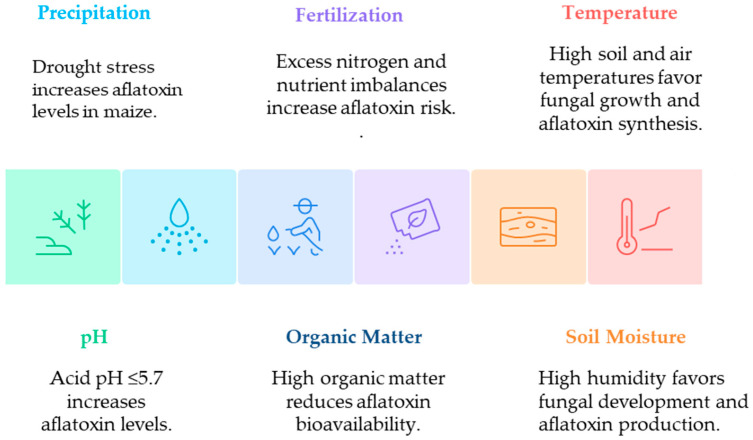
Environmental and edaphic factors influence the infection, development, and spread of *A. flavus* and aflatoxin production.

Several environmental and edaphic factors influence the infection, development, and spread of *A. flavus* and the subsequent AF production (Figure 3). Abiotic factors such as high soil and/or air temperature, drought stress, water activity, and humidity have been shown to greatly influence the AFs’ biosynthetic pathway in maize across different agroecological regions owing to year-to-year climatic conditions [67]. The impact of CC may vary by region and season. In most of Mexico, maize is grown under rainfed conditions (without irrigation), making plants more vulnerable to variability in temperature and precipitation patterns during the sensitive periods of flowering and grain development [52]. Soil composition and physicochemical properties significantly influence AF production and persistence in agricultural soils. The texture of clay loam soils has been associated with higher *Aspergillus* populations and AF production capacity than that of sandy loam soils [68]. These soils are distributed in different ways throughout the country, with variations in their properties and characteristics (https://paot.org.mx/centro/ine-semarnat/informe02/estadisticas_2000/informe_2000/index.htm, accessed on 31 July 2025). The nitrogen source is closely related to AF production, as some substrates, such as ammonium salts, favor AF production, whereas others, such as sodium nitrate, do not [69]. Soil pH plays a crucial role; at an acidic pH below 5.7, higher AF levels have been observed in a nitrate-based medium compared to those in alkaline soils above pH 7.2 [69]. Higher soil organic matter (SOM) levels reduce AFs’ bioavailability [70], and the relationship between SOM and AF accumulation is complex, as environmental factors, such as rainfall and vegetation cover during different crop growth stages, also play a crucial role.

In relation to the above, *A. flavus* tends to be present in high quantities and in all types of materials, such as dust, grains, and seeds, where it proliferates when it finds the right conditions. Therefore, grain storage should be carried out in well-constructed silos to prevent moisture migration, which in turn limits AF production [71]. However, storage conditions in economically less developed countries are not ideal (no metal silos or poorly designed silos, leaky roofs or dirt floors, and outdoor drying) [72].

### 3.3. Prevalence of Aflatoxins in Maize-Producing Regions in Mexico

Recent studies in Veracruz estimated that almost 70% of the population consumed AFs at levels above the recommended level [73]. A population-based study in eastern and southern Mexico revealed widespread exposure to AFB1, particularly among older adults, men, and rural residents [74]. Alarmingly, AF levels as high as 2630 µg/kg have been reported in Mexico and Central America between 2017 and 2021 [71]. Even more alarming, in 2020, AF levels as high as 4020 and 4405 µg/kg were detected in the northeast and southeast regions of Mexico, respectively [53]. These levels are comparable to those in Guatemala, which could contribute to the increased burden of liver cancer in these regions [75]. In dairy products, AFM1 concentrations exceeding the regulatory limit of 0.5 µg/kg have been found in some milk and artisanal cheese samples [76]. Widespread contamination of maize products in Mexico may contribute to hepatocellular carcinoma [77,78]. Table 6 presents a review of studies on the presence of AFs in maize grains and their byproducts, updated and expanded from Odjo et al. (2022) [71].

Research findings on cereals and roots (cassava) show that AFs remain prevalent in Latin American and Caribbean countries [71,72,89,90,91]. Despite the implementation of mitigation strategies, concerns remain regarding the risks to human health due to high consumption of staple foods. Therefore, government and institutional interventions are essential for developing sustainable strategies to prevent food contamination and protect public health [89].

## 4. Co-Occurrences of Aflatoxins and Fumonisins

### 4.1. Factors Associated with Co-Occurrence

The occurrence and co-occurrence of AFs and FUMs in maize have been observed in Mexico (Table 7), Central America [71,90], and South America [72,91,92]. The interaction between *A. flavus* and *F. verticillioides* involves chemically mediated competition, with AFs and FUMs serving as their main competitive metabolites [93]. These pathogenic fungi can infect and contaminate food with their mycotoxins, sharing the same ecological niche, where both fungi can colonize the same maize grains. In mixed infections, *F. verticillioides* is reported to be an opportunistic pathogen compared to other genera, such as *A. flavus* and *Penicillium* spp. [94]. Homologs of multiple FUM genes have been identified in various *Aspergillus* species [95]. Moreover, the presence of more than one mycotoxin in cereals has been reported to have additive or synergistic effects [96]. The presence of *A. flavus* and *F. verticillioides* does not necessarily imply the presence of mycotoxins, as many substrate and environmental factors determine their production. Similarly, the absence of any visible signs of the fungus does not guarantee that the grain is free of toxin, as the fungus may have been eliminated at some point in the process, but the AFs and FUMs formed could still be present in the grain. Therefore, the toxigenic profile of contaminated maize depends not only on the dominant pathogenic species but also on the presence and activity of other species, even if they are present in smaller proportions [97]. This situation is worsened by CC, crop stress, and poor agricultural practices, which pose food safety and security challenges [11,98,99].

### 4.2. Health, Food Safety, and Trade Implications

Recently, studies in southern and eastern Mexico and El Salvador found an association between maize and maize tortilla consumption and serum levels of AFB1-lysine albumin adduct (AFB1-lys). This link appears to be primarily due to the intake of homemade masa maize tortillas [90,105]. In contrast, a correlation was observed between urinary FUMB1 levels and maize tortilla intake in Mexican women [106]. To our knowledge, no studies on mycotoxin co-exposure with maize consumption have been published in the Mexican population, although an assessment of mycotoxin risk through maize tortilla intake has been conducted. This assessment showed that 70% of the population consumed more AFB1 than the recommended dose by the Joint FAO/WHO Expert Committee on Food Additives (1 ng/kg per day), while less than 5% consumed FUMB1 due to its low presence and levels in maize tortillas [73]. Another study in northeastern Mexico found that over half (57%) of the urine samples from 106 individuals had detectable AFM1 and FUMB1 levels. The same study also reported average concentrations of 5.3 µg/kg and 800 µg/kg of AFs in maize-derived foods, suggesting that co-exposure is common in this region [107].

**Figure 4:**
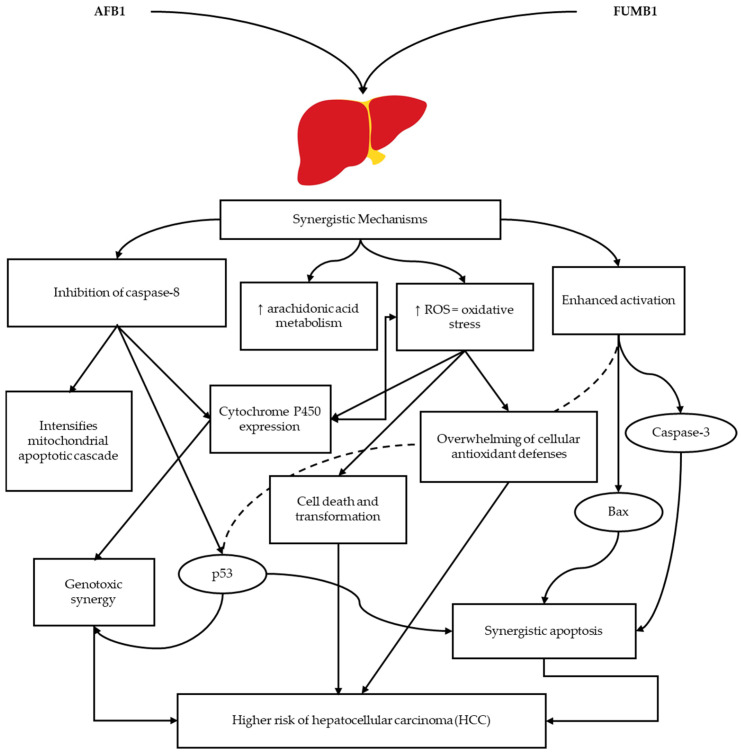
Convergent effects and synergy of aflatoxin B1 (AFB1) and fumonisin B1 (FUMB1): The synergistic mechanisms induced by combined exposure include increased production of reactive oxygen species (ROS), heightened oxidative stress, and activation of p53, Bax, and caspase-3-dependent apoptotic pathways. Furthermore, caspase-8 inhibition by FUMB1 has been shown to enhance the mitochondrial apoptotic pathway, contributing to a heightened risk of hepatocellular carcinoma (HCC) compared to exposure to AFB1 or FUMB1 alone.

A synergistic interaction between AFB1 and FUMB1, inducing cell apoptosis, has also been reported [108,109]. In HepG2 cells, immunocytochemical analysis of AFB1 and FUMB1 exposure showed a synergistic relationship with the expression of apoptosis-related proteins (Bax, Caspase 3, and p53). This synergistic pro-apoptotic activity is caused by different mechanisms owing to the expression of the antagonist caspase-8 [108]. Furthermore, a synergistic interaction toward genotoxicity in BRL-3A cells was suggested, which included an increase in arachidonic acid metabolism, cytochrome P450 activity, p53 levels, and reactive oxygen species (ROS) levels [109]. Therefore, co-exposure synergistically increases the properties of hepatocellular cancer, and this mixture may increase the toxic effects and lead to a more significant risk factor than exposure to the chemicals alone (Figure 4) [110].

### 4.3. Use of Biomarkers to Assess Exposure to Mycotoxins

The use of specific biomarkers for AFB1 and FUMB1 is a valuable tool for assessing individual mycotoxin exposure in epidemiological studies. Validated biomarkers such as AFB1-lys in serum, AFM1 and AF-N7-Gua in urine, T-DON, FUMB1, and phosphorylated sphingoid bases—offer more precise exposure estimates [111]. Nevertheless, the use of these instruments in longitudinal studies in Mexico is hindered by substantial methodological and logistical limitations in processing and storing biological samples in large-scale, long-term studies [112]. Despite these challenges, there is an opportunity to develop biomarkers using advanced methods and an approach that incorporates environmental information (mycotoxin content in food) and individual characteristics (diet, metabolism, and comorbidities) to generate solid evidence of the long-term effects of co-exposure to mycotoxins in the Mexican population.

## 5. Strategies to Mitigate Aflatoxin and Fumonisin Contamination

### 5.1. Sustainable Agricultural Practices

Specific agronomic practices in maize production may depend on the region to control *A. flavus* and *F. verticillioides*, especially by mitigating water stress, insect damage, and lowering crop susceptibility (Table 8). The evolution of integrated production management practices takes a holistic and sustainable approach, incorporating site-specific technologies and practices to optimize yields, reduce inputs, and ensure long-term environmental and economic sustainability [59].

### 5.2. Genetic Resistance in the Maize Plant

Improved germplasm is particularly needed to ensure maize yield and make it more resilient to CC [120]. Landrace or improved maize varieties must be well adapted to the areas where they are planted, with tolerance to drought, heat, insects, and pathogens [52]. Broad performance adaptation is essential to respond to global CC, the vagaries of spatial heterogeneity within farmers’ fields, the effectiveness of managing production inputs, and unpredictable seasonal and temporal climate variability [121]. Farmers best protected from mycotoxins because of CC are those with access to a steady stream of new cultivars bred for current climate conditions. However, most maize inbred lines were developed in a climate different from the current one (Table 2), which puts farmers at risk of mycotoxin contamination and crop failures. To reduce these risks, the effectiveness of regional and international germplasm exchange platforms must be improved [116,117,118]. Table 9 describes new CIMMYT tropical maize inbred lines with multiple tolerance/resistance to abiotic (high temperature, drought, low nitrogen use) and biotic stresses (ear rot and major foliar diseases), that were developed from crosses between elite lines and may be promising sources for in situ testing resistance to *A. flavus* and *F. verticillioides* infection and AF and FUM accumulation (https://www.cimmyt.org/resources/seed-request/, accessed on 20 June 2025). Nevertheless, the improvement component of adaptation strategies should focus on improving local farmers’ breeding practices. The desired outcome is a segmented maize seed sector characterized by both landraces (breeding) and hybrids [122,123].

**Table 9:** Novel inbred lines of tropical maize with multiple resistance/tolerance to biotic and abiotic factors, which may be promising sources for in situ testing resistance to *A. flavus* and *F. verticillioides* infection and AF and FUM accumulation.

Inbred Line ^1^	Germplasm Source ^2^	Inbred Line ^1^	Germplasm Source ^2^
**Drought** **-tolerant, resistant to ear rot and major foliar diseases, tropical white for Latin America**			
CML515	CML247/IR	CML576	CLFAWW11/CML494
CML549	CML498/CLRCW36	CML596	CL04325/CML401
CML550	P25HSRRS	CML600	CLRCW88/CLRCW96
CML552	CML495/CML401	CML601	CLRCW79/CLRCW98
CML553	CML264/CLRCW41	CML636B	CML269/CL02221
CML554	CML491/CLQRCWQ13	CML638A	CLG2305/CML401
CML555	H132	CML639B	CML555/CLQRCWQ121
CML556	CML502/CLQRCWQ26	CML640B	CL02221/CLRCW72//CML556
CML557	CML176/CML264		
**Drought-tolerant, resistant to ear rot and major foliar diseases, yellow for Latin America**			
CML551	P27FRRS	CML598	CML413/CML287
CML575	CML451/CLRCW29	CML599	P390AM
CML577	CML454/CML451	CML602	CLRCY040/CML451
CML597	CML285/CL00356	CML637B	CML451/CML551
**Drought-tolerant, resistant to ear rot and major foliar diseases, white for Eastern and Southern Africa**			
CML569	LAPOSTASEQ/CML395	CML609A	CML495/PHG39
CML570	LAPOSTASEQ/CML444	CML610A	CKL05017/LAPOSTASEQ
CML607B	LAPOSTASEQ/CML395	CML618B	CML384/(MBR/MDR
CML608B	ZM521B/LAPOSTASEQ	CML620B	CML543/(CML444//CML395///DTPW
**Drought-tolerant and provitamin A-enhanced tropical mid-altitude, yellow for Southern Africa**			
CML628B	KUICAROTENOIDSYN/CML297///KUI3/SC55		
CML629B	CML488/(BETASYN)BC1//G9BTSR///ATZT-VC82		
CML630B	CLQRCWQ97/KUICAROTENOIDSYN///KU1409		

^1^ Inbred line code: CIMMYT Maize Line (CML) https://www.cimmyt.org/resources/seed-request/ (accessed April 2025). ^2^ Short pedigree from which CML was formed.

### 5.3. Biological Control

#### 5.3.1. Atoxigenic Strains of *A. flavus*

The concept of non-aflatoxin-producing strains of *A. flavus* was initiated in the late 1980s to reduce AF contamination in cotton crops in Arizona [124]. Since then, the viability of biocontrol has been demonstrated in commercial applications for other crops, including maize, peanuts, sorghum, pistachios, almonds, and figs. The new generation of biocontrol products contains strains that do not produce AFs or cyclopiazonic acid (CPA), known as non-toxigenic strains (atoxigenic strains: Table 10). The biocontrol mechanism of atoxigenic strains occurs by competitive exclusion, which indicates competition for the same resources (nutrients, water, and space) that toxigenic strains would use; in this process, the atoxigenic strains displace the toxigenic strains present in treated agricultural soils [125]. Other potential mechanisms include the degradation of AFs by using them as a carbon source [126], thigmoregulation by intraspecific inhibition that prevents or regulates the low expression of AFs [127], and chemodetection through excretory products and volatile organic compounds secreted by atoxigenic strains [128]. Biocontrol formulations with autochthonous atoxigenic strains have superior competitive ability against other indigenous microorganisms owing to local resources, adaptation to the environment, cropping systems, and climatic and soil conditions [129]. Furthermore, native fungi as active ingredients in products allow for faster regulatory approval than exotic fungi [130].

**Table 10:** Biological control products based on atoxigenic strains of *A. flavus* are currently marketed to reduce aflatoxin levels [130].

Commercial Product	Strain Name	Isolation Source	Place of Application	Use in Crops
AF36 Prevail^® 1^	AF36	Cottonseed	United States	Cotton, maize, fig, almond, pistachio
Afla-Guard^® 2^	NRRL21882	Peanut	United States	Maize, peanut, almond, pistachio
Aflasafe^™ 3^	Ka16127, La3279, La3304, Og0222	Maize soils	Nigeria	Maize, peanut
Aflasafe KE01^™^	C6-E, C8-F, E63-I, R7-H	Maize soils	Kenya	Maize
Aflasafe SN01	M2-7, M21-11, Ms14-19, Ss19-14	Maize and peanut soils	Senegal, Gambia	Maize, peanut
Aflasafe BF01	M011-8, G018-2, M109-2, M110-7	Maize and peanut soils	Burkina Faso	Maize, peanut
Aflasafe GH01	GHG079-4, GHG083-4, GHG321-2, GHM174-1	Maize and peanut soils	Ghana	Maize, peanut, sorghum
Aflasafe GH02	GHM511-3, GHM109-4, GHM001-5, GHM287-10	Maize and peanut soils	Ghana	Maize, peanut, sorghum
Aflasafe TZ01	TMS199-3, TMH104-9, TGS364-2, TMH 30-8	Maize and peanut soils	Tanzania	Maize, peanut
Aflasafe TZ02	TMS64-1, TGS55-6, TMS205-5, TMS137-3	Maize and peanut soils	Tanzania	Maize, peanut
Aflasafe MWMZ01	GP5G-8, GP1H-12, MZM594-1, MZM029-7	Maize and peanut soils	Mozambique	Maize, peanut
Aflasafe MWMZ01	MW199-1, MW097-8, MW246-2, MW238-2	Maize and peanut soils	Malawi	Maize, peanut
Aflasafe MZ02	GP5G-8, MZG071-6, MZM028-5, MZM250-8	Maize and peanut soils	Mozambique	Maize, peanut
Aflasafe MW02	MW258-6, MW332-10, MW248-11, MW204-7	Maize and peanut soils	Malawi	Maize, peanut
Aflasafe ZM01	110MS-05, 38MS-03, 46MS-02, 03MS-10	Maize and peanut soils	Zambia	Maize, peanut
Aflasafe ZM02	31MS-12, 12MS-10, 47MS-12, 64MS-03	Maize and peanut soils	Zambia	Maize, peanut
AF-X1^® 4^	MUCL54911	Maize cob	Italy	Maize
FourSure™ ^5^	TC16F, TC35C, TC38B, TC46G–FFDCA	Maize fields	Texas	Maize

^1^ AF36 marketed by the Arizona Cotton Research and Protection Council (ACRPC). ^2^ Afla-Guard marketed by SYNGENTA. ^3^ Aflasafe marketed by the International Institute of Tropical Agriculture (IITA). ^4^ AF-X1 marketed by CORTEVA. ^5^ FourSure marketed by the Texas Maize Producers Board.

#### 5.3.2. Soil Microbiome

Stress-tolerant soil microbial communities and rhizomicrobiomes play crucial roles in mitigating mycotoxins. Key genes that regulate the assembly and composition of the rhizosphere microbiome have been identified in plant genomes, influencing root morphology, metabolism, exudates, nutrient uptake, and immune responses [131]. A study conducted on landrace maize grown under a polyculture system on plant growth-promoting rhizobacteria (PGPR) described the diversity, functionality, and detection of potential rhizobacteria for further development of biofertilizers and targeted biocontrollers as biotechnology for sustainable agriculture [132]. Another metagenomics study on the rhizosphere of teosinte, landraces, and improved maize lines to explore the association between maize accessions and rhizosphere microbial assemblages found the highest diversity of the rhizobacterial community, providing new insights into integrating soil nutrient availability and improving microbial co-evolution in maize breeding [133]. The evolutionary background behind the diversity of various mycotoxin chemotypes in fungi to produce either all or only some mycotoxins and the preference for a specific habitat can be determined by comprehensively analyzing the genomes of several strains with different biosynthetic profiles of relevant AFs and FUMs, both at the genetic and analytical levels [134].

## 6. Conclusions

The occurrence of both AFs and FUMs in Mexican maize poses a serious threat to food safety and public health. Despite decades of research, monitoring, and mitigation strategies, consumer protection remains insufficient. The co-occurrence of AFs and FUMs, particularly in southern and eastern Mexico, has been linked to synergistic toxic effects that increase the risk of hepatocarcinogenesis. AF levels exceeding 4000 µg/kg have been reported, which is well above the regulatory limits. Although AFs are regulated in Mexico, no official limits exist for FUMs, highlighting a critical policy gap in this area. Environmental and soil conditions are caused by negative effects of CC, such as drought, rising temperatures, pH, and organic matter, further influencing fungal toxin production. Sustainable agricultural practices, such as crop diversification, integrated pest management, efficient irrigation, and conservation agriculture, can reduce AF incidences by improving crop and soil health. Using native, atoxigenic strains of *A. flavus* as a biocontrol strategy is promising because it displaces the toxigenic strains present in treated agricultural soils through competitive exclusion.

## 7. Future Directions

### 7.1. Other Significant Mycotoxins Found in Maize

The effects of CC in Mexico due to high temperatures and drought are expected to be more conducive to infection by *Aspergillus* section *Flavi* species, resulting in subsequent AF contamination. Meanwhile, warm conditions and abundant rainfall could be more favorable for infection by *Fusarium* spp. [135] as well as for the fungi *Penicillium* spp. and *Stenocarpella maydis* (formerly *Diplodia*), which produce Ochratoxin A (OTA) and Diplodiatoxin or Diplodiol (Dpl tox), respectively [16,136]. Furthermore, conditions with relatively cool temperatures and frequent rainfall during the flowering and ripening periods are more favorable for *F. graminearum*, which produces the mycotoxins deoxynivalenol (DON) and zearalenone (ZEA) [137]. Recent studies of maize in Mexico and South America during 2023 revealed an increase in the prevalence and contamination of ZEA and DON [22]. Certain mycotoxigenic fungal species are expected to readily acclimatize to new conditions and could become more aggressive pathogens. Furthermore, abiotic stress factors resulting from CC are expected to weaken the resistance of host crops, rendering them more vulnerable to fungal disease outbreaks [98]. In this sense, future research should study the occurrence and co-occurrence of DON, ZEN, and FUMs in temperate and subtropical maize regions, and FUMs and AFs in subtropical and tropical climates, in addition to OTA and Dpl tox.

### 7.2. Modern Strategies to Optimize Maize Breeding

The biological breeding strategies 3.0 stage mainly include marker-assisted selection, genomic selection, genetic engineering, haploid induced breeding, gene editing, and synthetic biology, which act as breeding accelerators and lead to maize improvement in different important traits, such as grain yield, grain quality, biotic and abiotic stress resistance and/or tolerance, and nitrogen use efficiency. Several promising intelligent breeding strategies in the next era of the 4.0 stage will improve maize production greatly for ensuring global food security [138]. These breeding strategies should include artificial inoculations with local isolates of ear rot in various environmental conditions with reports of high mycotoxin incidence ([27,139], which allow simultaneous evaluation of tolerance to biotic (pathogens, insects, and weeds) and abiotic (high temperatures and drought) stresses to provide suitable pressure in the effective identification of resistance genes useful for modern breeding programs. Mexican maize landraces, which are adapted to diverse agroecological conditions, are underutilized genetic resources that may offer resistance to AF and FUM contamination. These should be identified and incorporated into breeding programs alongside elite lines to develop resilient maize varieties.

### 7.3. Improving Microbial Understanding in Biocontrol Development

Soil is the main reservoir of *A. flavus*, and atoxigenic strains are isolated from tropical and subtropical latitudes, where they commonly infect crops [140]. Understanding genetic diversity and how atoxigenic strain populations change over time after their release into the environment is important [141]. Similarly, it is crucial to measure the number of years in which atoxigenic strains should be applied [142]. It is also important to understand how environmental factors and soil movement during tillage operations influence the distance and dispersion of biocontrol agents to determine whether the treatment is effective [143]. To address these challenges, future research should prioritize microbiome engineering (metagenomics, metatranscriptomics, metabonomics, and metaproteomics), precision agriculture, and the development of climate-resilient microbial strains [144]. In this regard, the development of associations with biocontrol agents is promising. In addition, these approaches may provide an avenue for the identification of metabolites that could serve as novel, effective, specific, and more environmentally friendly fungicides against *Aspergillus* and *Fusarium* [145].

### 7.4. Aflatoxins’ Predictive Risk Models in Maize

Mycotoxin models capable of predicting AF and FUM outbreaks in cultivated maize are key tools for addressing the impact of CC on plant-pathogen interactions. Several predictive models have been developed worldwide [98], based on mechanistic and artificial intelligence (AI) algorithms [146,147]. However, environmental data provide only marginal benefits for predicting climate adaptation [148]. This is due to abiotic and biotic stress factors [149,150]. Recent predictive models have incorporated soil properties using a geospatially dynamic approach to quantify the contributions of these factors to historical mycotoxin outbreaks [151,152]. These models demonstrated that, in addition to pre- and post-planting weather factors, soil properties are significantly correlated with AF and FUM contamination at harvest and should be included in future models.

### 7.5. Clinical and Epidemiological Studies on Mycotoxins

In Mexico, a high prevalence of individuals with detectable levels of circulating AFB1 has been identified, and it has been documented that circulating levels of AFB1 are associated with maize consumption. However, the cross-sectional design and single measurement of the AFB1 biomarker (AFB1-lys) limit causal inference and the assessment of seasonal variations in exposure to mycotoxins. It is imperative to characterize exposure over time through prospective studies in regions of higher exposure and to expand population coverage in national surveys. A specific assessment of high-risk groups, including children, women, farm workers, and maize producers, such as those engaged in mill and tortilla factory work (i.e., Tortillerias), will facilitate the identification of risk patterns that vary according to specific characteristics. Concurrently, scientific evidence has documented that co-exposure to FUMB1 increases the toxicological impact, thereby reinforcing the necessity to assess simultaneous exposures in population contexts and to improve the detection of biomarkers in blood or urine. In this context, future research should consider longitudinal designs, the inclusion of high-risk populations, the analysis of tumor tissues in areas of higher exposure, and the evaluation of mixtures with other mycotoxins. Consequently, it is imperative to enhance the technical capacity for population monitoring and implement validated biomarkers for co-exposure.

## Figures and Tables

**Table 1:** Upper permissible limits for aflatoxins and fumonisins in human and animal consumption in Mexico.

Commodity	Upper Limit µg/kg	
	AF B1	FUM B1 + B2
All products destined for humans	20 ^1^	4000 ^4^
Nixtamalized maize flour and masa for tortillas	12 ^2^	2000 ^4^
Milk	0.5 ^3^	N/A ^5^
All products destined for poultry	100 ^1^	N/A
Swine	200 ^1^	N/A
Cattle	300 ^1^	N/A

^1^ NOM-188-SSA1-2002. ^2^ NOM-247-SSA1-2008; NMX-FF-034/1-SCFI-2002. ^3^ NOM-243-SSA1-2010. ^4^ FDA Guidance for the Industry. ^5^ No specific information was available.

**Table 2:** Tropical maize inbred lines resistant to *Aspergillus flavus* and aflatoxin production.

Inbred Line Code	Sourced Institution	Ref.
Mp313E, Mp 420, Mp 715, Mp717, Mp 718 and Mp719	Mississippi State University, USA	[31,32,33,34,35]
CML176, CML269 and CML322	CIMMYT ^1^ and Texas A&M University, USA	[36]
GT-601, GT-602 and GT-603	University of Georgia Coastal Plain, USA	[37,38]
CML348, NC388, NC400, NC408 and NC458	CIMMYT and North Carolina State University, USA	[39]
CML52, CML69, GEMS-0005, Hi63, Hp301 and M37 W	CIMMYT and University of Georgia, USA	[40]
Tx736, Tx739, Tx740, Tx741, Tx777, Tx779, Tx780 and Tx782	Texas A&M and Texas AgriLife Research Maize, USA	[41,42]
TZAR101, TZAR102, TZAR103, TZAR104, TZAR105 and TZAR106	IITA ^2^, West and Central Africa	[43]
CML247, CML444 and CML495	CIMMYT and University of Nairobi, Kenya, and South Africa	[44,45]
CML247 and CML495	CIMMYT, Southern Mexico	[27]

^1^ International Maize and Wheat Improvement Center. ^2^ International Institute of Tropical Agriculture.

**Table 4:** Description of the parameters comparing intensive and agroecological practices to achieve sustainable maize production, according to Yamini et al. (2025) ^1^ [59] and their relationship to mycotoxin contamination, especially aflatoxins (AFs) and fumonisins (FUMs).

Parameter	Intensive Practices	Agroecological Practices	Relationship with Mycotoxin Contamination
Soil fertility	Heavy reliance on chemical fertilizers, leading to nutrient imbalances and soil degradation.	Combines organic and inorganic inputs, promoting balanced nutrition and improved soil structure and fertility.	Favorable, balanced nutritional conditions improve plant defenses; e.g., optimal nitrogen application reduces mycotoxin contamination.
Nutrient management	Generalized fertilizer application without soil testing, often resulting in inefficiencies.	Site-specific nutrient management based on scientific assessments, such as soil health cards, for optimal nutrient use.	Periodic soil testing helps determine the specific nutritional needs of the crop and allows for targeted fertilizer application.
Water management	Inefficient irrigation methods, leading to water wastage and salinization.	Promotes efficient techniques like micro-irrigation, drip systems, rainwater harvesting, and scheduling based on crop needs.	Maintaining optimal soil moisture levels creates unfavorable conditions for mycotoxin-producing fungi and improves the resilience of drought-tolerant maize.
Crop diversification	Monocropping dominates, increasing vulnerability to pests, diseases, and market risks.	Encourages diverse cropping systems, including rotations and intercropping with cereals, pulses, and horticultural crops.	Crop rotation disrupts the life cycle of mycotoxigenic fungi and improves microbial diversity.
Resource use efficiency	Overuse of inputs like water, fertilizers, and pesticides, reducing long-term productivity.	Focuses on precise and judicious use of inputs to enhance efficiency and reduce costs and environmental impact.	Pesticides reduce pest populations associated with mycotoxin contamination. However, excessive use reduces the number of natural enemies and can lead to pesticide resistance.
Pest and disease management	Sole reliance on chemical pesticides, leading to resistance and ecological imbalance.	Advocates integrated pest management (IPM) and agroecological pest management (APM), combining biological, cultural, and chemical controls to manage pests sustainably.	IPM or APM approaches can significantly reduce mycotoxin contamination and improve crop quality.
Conservation agriculture (CA)	Rarely adopted, leading to soil erosion and loss of organic matter.	Incorporates practices like minimum tillage, residue retention, and crop rotations to conserve soil and water resources.	CA promotes soil health and creates a less favorable environment for *Aspergillus* and *Fusarium.*
Yield and productivity	Short-term yield gains but declining productivity over time due to resource degradation.	Maintains or improves yields sustainably through holistic management of inputs, pests, and environmental factors.	High-yield practices help reduce plant stress and the risk of fungal infection.
Economic viability	High input costs and diminishing returns in the long run.	Reduces input costs through efficient practices, improving profit margins for farmers.	Cost-effectiveness and economic incentives are crucial for adopting control methods across different agricultural sectors.
Environmental impact	Contributes to environmental issues like water pollution, greenhouse gas emissions, and loss of biodiversity.	Minimizes environmental footprint by reducing reliance on synthetic inputs and adopting eco-friendly practices.	The different agroecological practices help prevent and reduce the conditions that favor the growth of mycotoxin-producing fungi in the field and during postharvest.

^1^ Source: https://doi.org/10.3389/fsufs.2025.1428687.

**Table 5:** Summary of major pests associated with aflatoxin and fumonisin contamination of maize.

Insect/Pathogen	Morphological Description	Habits and Pest Structures	Critical Period	Ref.
Budworm: *Spodoptera* *frugiperda* (Lepidoptera: Noctuidae)	The adult is a dark gray moth with a white spot on the wings and lays its eggs on the underside of leaves. After six larval stages, the grayish-brown maggot measures 3 cm.	The cannibalistic larva is a bud and leaf chewer. Before pupating, it falls to the ground and may feed on tender stalks.	Vegetative	[62]
Corn earworm: *Helicoverpa zea* (Lepidoptera: Noctuidae)	The adult is a brown moth, laying eggs at the R1 stage. The first instar larva is gray with a black head, and in the last instar (sixth) it is pink.	The larva feeds on stigmas, silk, and cob. Noctuid moths tend to fly hundreds of miles in search of food.	Reproductive	[63]
Maize weevil: *Sitophilus zeamais* (Coleoptera: Curculionidae)	The adult is black, 3.5 mm, with a long proboscis, and lays its eggs inside the grain. The larvae are creamy white. Between 6 and 7 generations are produced per year.	The flying adult and larva feed on the grain, affecting seed germination during feeding and facilitating the introduction of *Aspergillus*.	Maturity and postharvest	[64]
Ear rot: *Fusarium verticillioides* (Telemorph: *Gibberella moniliformis*)	The fungus produces ovoid microconidia in chains and macroconidia in purplish pink aerial mycelium.	The chlamydospores survive in the plant debris. With the first rains or irrigations, the conidia germinate and are spread by the wind to infect several points distributed in the ear and/or asymptomatic grains.	Reproductive and Maturity	[65]
Ear rot: *Aspergillus flavus* (Teleomorph: *Petromyces flavus*)	The fungus produces purplish-brown-green conidiophores in aerial mycelium.	Sclerotia survive in the soil under warm weather and drought conditions. Airborne and insect dispersal of conidia are associated with infection.	Harvest and storage	[66]

**Table 6:** Studies on the presence of aflatoxins in maize and its byproducts in Mexico ^1^.

States	Maize Product	Number of Samples (*n*)	AFB1 µg/kg (Maximum Level Found)	Year ^4^	Ref.
Tamaulipas and Campeche	Grain	1479	4405	2025	[53]
Tamaulipas	Grain	35	955	2005	[79]
Nayarit	Grain	49	21	2021	[80]
Aguascalientes	Grain	11	26	2013	[81]
Puebla and Tlaxcala	Grain	80	12	2024	[82]
San Luis Potosí	Nixtamalized grain	327	287	2018	[83]
México city	Nixtamalized grain	88	16	2019	[84]
Veracruz	Tortilla local market	120	22	2019	[73]
Mexico City	Tortilla local market	396	20	2011	[85]
Veracruz	Popcorn	30	26	2020	[86]
Chiapas	Pozol ^2^	111	21	2004	[87]
Mexico	Domestic pet foods (dog and cat) ^3^	35	72.4	2001	[88]

^1^ Very limited information is available on this topic. ^2^ A traditional drink made from fermented maize, including cocoa. ^3^ Different trademarks obtained from different department stores. ^4^ Publication year.

**Table 7:** Optimum temperature, precipitation or drought, grain moisture, and Mexican states with tropical and subtropical maize-growing regions, where aflatoxin and fumonisin occurrences and co-occurrences have been reported ^1^.

Mycotoxin	Temperature (°C)	Rainfall/Drought	Grain Moisture (%)	Reporting States	Reference
Aflatoxins	30–36	Drought	≥14	Sonora, Tamaulipas, Campeche Veracruz, Chiapas, Yucatán, and Guerrero.	[53,100,,101]
Fumonisins	28–34	Rainfall	≥18	Puebla, Guanajuato, Jalisco, Nayarit, Sinaloa, Coahuila, Chihuahua, Veracruz, and Chiapas.	[102,103]
Aflatoxins and Fumonisins	30–34	Drought and rainfall intervals	18–25	Veracruz and Chiapas	[73,,102,104]

^1^ Very limited information: The available information focuses on the ideal conditions for the development of pathogens and their mycotoxins, but does not provide details on the movement of grain within the country. The geographical movement of maize for distribution and consumption remains unknown.

**Table 8:** Management strategy for insects (*S. frugiperda*, *H. zea*, *S. zeamais*) and pathogens (*F. verticillioides*) that provide entry sites for *A. flavus* are associated with aflatoxin contamination.

Practice	*Spodoptera* *frugiperda*	*Helicoverpa* *zea*	*Sitophilus* *zeamais*	*Fusarium* *verticillioides*	*Aspergillus* *flavus*
Genetic	Conduct pilot tests with several commercial hybrids and/or landraces with good adaptation to have genetic variation and to serve as a protective barrier to prevent the spread of pests.				Maize with excellent ear coverage and tolerance to drought, high temperatures, and insects significantly reduces fungal infestation and aflatoxin production (Table 9).
Agronomical	Soil removal before planting to expose larvae and pupae to the sun.	Weed control and densities ≤75 thousand plants/ha.	Dry and store grain at humidity ≤16%.	Sow pathogen-free seed.	Harvest when grain moisture is ≤25%. Adjust the threshing machine to avoid grain breakage. Dry and store grain at moisture ≤13% [71].
Biological	*Campoletis sonorensis* and *Cotesia marginiventris* [113]. Phero-SF pheromones [114].	*Trichogramma* spp., *Hippodamia convergens* and *Bacillus thuringiensis* [115].	*Metarhizium* *anisopliae* and *Beauveria bassiana* [116].	*Trichoderma asperellum* [117]. *Bacillus* spp. [118].	Use of atoxigenic strains of *A. flavus* (Table 10).
Chemical	Spinetoram (Palgus-Dow). Dosage: 75–100 mL/ha. Flubendiamide (Belt-Bayer): Dosage: 100–125 mL/ha. Application: Direct spraying to leaves and buds when 30–40% of plants with perforated leaves, larvae, or droppings are observed.	Chlorantraniliprole (Coragen-FMC). Dosage: 200–250 mL/ha. Avermectin (Denim-Syngenta). Dosage: 100–200 mL/ha. Application: Make three applications to the foliage at 10-day intervals. Start the first one at flowering.	Phosphine (Phostoxin-Degesch) Dosage: 3 tablets/t. Application: Must gas for 72 h. Then ventilate the area for 24 h. Repeat after 3 months.	Seed treatment: Fludioxonil + metalaxyl (Maxim-Syngenta). Dosage: 100 mL/kg seed. It is recommended to mix with systemic insecticides such as azoxystrobin and trifloxystrobin [119].	

## Data Availability

The data presented in this study are available in this article.
