# Correction: Muñoz-Zavala et al. Aflatoxins in Mexican Maize Systems: From Genetic Resources to Agroecological Resilience and Co-Occurrence with Fumonisins. *Toxins* 2025, *17*, 531

**DOI:** 10.3390/toxins17120582

**Published:** 2025-12-04

**Authors:** Carlos Muñoz-Zavala, Obed Solís-Martínez, Jessica Berenice Valencia-Luna, Kai Sonder, Ana María Hernández-Anguiano, Natalia Palacios-Rojas

**Affiliations:** 1 Colegio de Postgraduados (COLPOS), Campus Montecillo, Texcoco 56264, Mexico; c.munoz@cgiar.org (C.M.-Z.); valencia.jessica@colpos.mx (J.B.V.-L.); 2 International Maize and Wheat Improvement Center (CIMMYT), Texcoco 56237, Mexico; k.sonder@cgiar.org; 3 National Institute of Public Health (INSP), Cuernavaca 62100, Morelos, Mexico; obed.solis@insp.edu.mx

## Error in Table

In the original publication [1], there was a mistake in Table 5 as published. “The chlamydospores survive in the plant debris.” in column three on the fifth row should be deleted. The corrected Table 5 appears below. The authors state that the scientific conclusions are unaffected. This correction was approved by the Academic Editor. The original publication has also been updated.

## Figures and Tables

**Table 5:** Summary of major pests associated with aflatoxin and fumonisin contamination of maize.

Insect/Pathogen	Morphological Description	Habits and Pest Structures	Critical Period	Ref.
Budworm: *Spodoptera* *frugiperda* (Lepidoptera: Noctuidae)	The adult is a dark gray moth with a white spot on the wings and lays its eggs on the underside of leaves. After six larval stages, the grayish-brown maggot measures 3 cm.	The cannibalistic larva is a bud and leaf chewer. Before pupating, it falls to the ground and may feed on tender stalks.	Vegetative	[62]
Corn earworm: *Helicoverpa zea* (Lepidoptera: Noctuidae)	The adult is a brown moth, laying eggs at the R1 stage. The first instar larva is gray with a black head, and in the last instar (sixth) it is pink.	The larva feeds on stigmas, silk, and cob. Noctuid moths tend to fly hundreds of miles in search of food.	Reproductive	[63]
Maize weevil: *Sitophilus zeamais* (Coleoptera: Curculionidae)	The adult is black, 3.5 mm, with a long proboscis, and lays its eggs inside the grain. The larvae are creamy white. Between 6 and 7 generations are produced per year.	The flying adult and larva feed on the grain, affecting seed germination during feeding and facilitating the introduction of *Aspergillus*.	Maturity and postharvest	[64]
Ear rot: *Fusarium verticillioides* (Telemorph: *Gibberella moniliformis*)	The fungus produces ovoid microconidia in chains and macroconidia in purplish pink aerial mycelium.	With the first rains or irrigations, the conidia germinate and are spread by the wind to infect several points distributed in the ear and/or asymptomatic grains.	Reproductive and Maturity	[65]
Ear rot: *Aspergillus flavus* (Teleomorph: *Petromyces flavus*)	The fungus produces purplish-brown-green conidiophores in aerial mycelium.	Sclerotia survive in the soil under warm weather and drought conditions. Airborne and insect dispersal of conidia are associated with infection.	Harvest and storage	[66]
